# The FGFR Family Inhibitor AZD4547 Exerts an Antitumor Effect in Ovarian Cancer Cells

**DOI:** 10.3390/ijms221910817

**Published:** 2021-10-06

**Authors:** Yu Ran Na, Jin Young Kim, Chang Ho Song, Mikyung Kim, Yen Thi Do, Tam Thuy Lu Vo, Eunsom Choi, Eunyoung Ha, Ji Hae Seo, So-Jin Shin

**Affiliations:** 1 Department of Gynecology and Obstetrics, School of Medicine, Keimyung University, Daegu 42601, Korea; ur3039@naver.com (Y.R.N.); songchanghomd@gmail.com (C.H.S.); yen94d@gmail.com (Y.T.D.); eunsom1113@naver.com (E.C.); 2 Department of Internal Medicine, Keimyung University School of Medicine, Daegu 42601, Korea; takgu@dsmc.or.kr; 3 Department of Biochemistry, Keimyung University School of Medicine, Daegu 42601, Korea; cjstk2227@nate.com (M.K.); volutam@gmail.com (T.T.L.V.)

**Keywords:** AZD4547, cancer stem cells, fibroblast growth factor, fibroblast growth factor receptor, ovarian cancer

## Abstract

The dysregulation of fibroblast growth factor (FGF) signaling has been implicated in tumorigenesis, tumor progression, angiogenesis, and chemoresistance. The small-molecule AZD4547 is a potent inhibitor of FGF receptors. This study was performed to investigate the antitumor effects and determine the mechanistic details of AZD4547 in ovarian cancer cells. AZD4547 markedly inhibited the proliferation and increased the apoptosis of ovarian cancer cells. AZD4547 also suppressed the migration and invasion of ovarian cancer cells under nontoxic conditions. Furthermore, it attenuated the formation of spheroids and the self-renewal capacities of ovarian cancer stem cells and exerted an antiangiogenic effect. It also suppressed in vivo tumor growth in mice. Collectively, this study demonstrated the antitumor effect of AZD4547 in ovarian cancer cells and suggests that it is a promising agent for ovarian cancer therapy.

## 1. Introduction

Ovarian cancer is the fifth leading cause of cancer-related death in the world and the most lethal gynecologic malignancy [1,2]. Approximately 70% of women with ovarian cancer are not diagnosed until the disease has progressed to an advanced stage [3]. Despite its high response rate to platinum-based drugs, ovarian cancer is characterized by high rates of resistance to chemotherapy and a high rate of relapse, which lead to progressively shorter durations of remission and ultimately a high rate of mortality [4,5]. Therefore, it is critical and urgent to find more effective treatment modalities for ovarian cancer.

Fibroblast growth factor receptors (FGFRs) are a family of receptor tyrosine kinases FGFR1–4 that respond to and transmit signals of FGFs and comprise 22 ligands [6,7]. The FGF/FGFR signaling pathway is involved in proliferation, differentiation, tissue modeling, and angiogenesis via gene amplification, overexpression, point mutations, or chromosomal translocations; these dysregulations can lead to the development and/or the progression of cancer [8]. The inhibition of FGF/FGFR signaling as a therapeutic approach for cancer is gaining momentum with several targeted kinase inhibitors currently under development [9]. The FGFR1 inhibitor SU5402 was shown to reduce the survival of breast, lung, and urothelial carcinoma cells [10,11,12]. Similarly, the FGFR1 and FGFR3 dual inhibitor PD173074 blocked cell cycle progression and caused apoptosis in lung, head and neck, breast, and urothelial carcinoma cells [11,13,14,15]. The FGFR1–3 inhibitor BGJ398 suppressed the migration of urothelial and bladder cancer cells [16,17].

Dysregulation of FGFR activity has also been reported in ovarian cancer [17]. The expression of FGFR4 was found to be elevated in serous ovarian carcinoma and was demonstrated to be significantly associated with poor overall survival [18].

AZD4547 is an FGFR1–3 selective inhibitor with a high potency against these receptors and a much lower potency against FGFR4 [19]. AZD4547 has been shown to promote favorable therapeutic outcomes against various FGFR-dysregulated cancers, including glioblastoma and lung, gastric, and endometrial cancer [20,21,22]. However, there is limited information about the antitumor effects and the mechanistic details of AZD4547 in ovarian cancer. In this study, we examined the antitumor effects and determined the mechanistic details of AZD4547 in various ovarian cancer cell lines. We demonstrated that AZD4547 suppressed the proliferation, survival, migration, and stemness of ovarian cancer cells. AZD4547 also inhibited angiogenesis in human umbilical vein endothelial cells (HUVECs). Finally, we confirmd the antitumor effect of AZD4547 in vivo in a mouse xenograft model.

## 2. Results

### 2.1. High Expression Levels of FGFR2 and FGFR3 Are Associated with a Low Survival Rate in Ovarian Cancer

**Figure 1:**
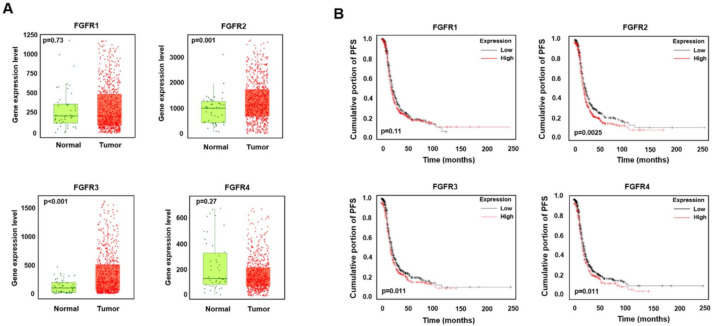
High expression of FGFR2 and FGFR3 is associated with low survival in ovarian cancer. (**A**) Box plot of gene expression between normal ovarian tissues and ovarian cancer tissues. Data were obtained from www.TNMplotter.com (accessed on 20 December 2020), which analyzed and visualized microarray data from NCBI Gene Expression Omnibus datasets. (**B**) Association between gene expression and progression-free survival. Data were obtained from www.KMplotter.com (accessed on 20 December 2020), which analyzed the data of patients with serous ovarian cancer from The Cancer Genome Atlas database. Affymetrix ID is valid: 207822_at (FGFR1), 203639_s_at (FGFR2), 204380_s_at (FGFR3), and 204579_at (FGFR4).

We investigated the expression levels of four FGFR isotypes (FGFR1, FGFR2, FGFR3, and FGFR4) in the open data from the TNM plotter database. We observed the upregulation of FGFR2 and FGFR3 expression in patients with ovarian cancer (Figure 1A). As expected, the patients with high FGFR2 and FGFR3 expression had low survival rates (Figure 1B). The patients with high FGFR4 expression also had low survival rates, although the FGFR4 expression level was not significantly different between normal and cancer tissues (Figure 1A,B). These data imply a possible role of FGFRs in ovarian cancer. Thus, we performed in vitro studies to determine the antitumor effects and mechanistic details of FGFRs in ovarian cancer using AZD4547, a small-molecule inhibitor of FGFR1–3.

### 2.2. AZD4547 Exerts Antiproliferative Effects in Ovarian Cancer Cells

**Figure 2:**
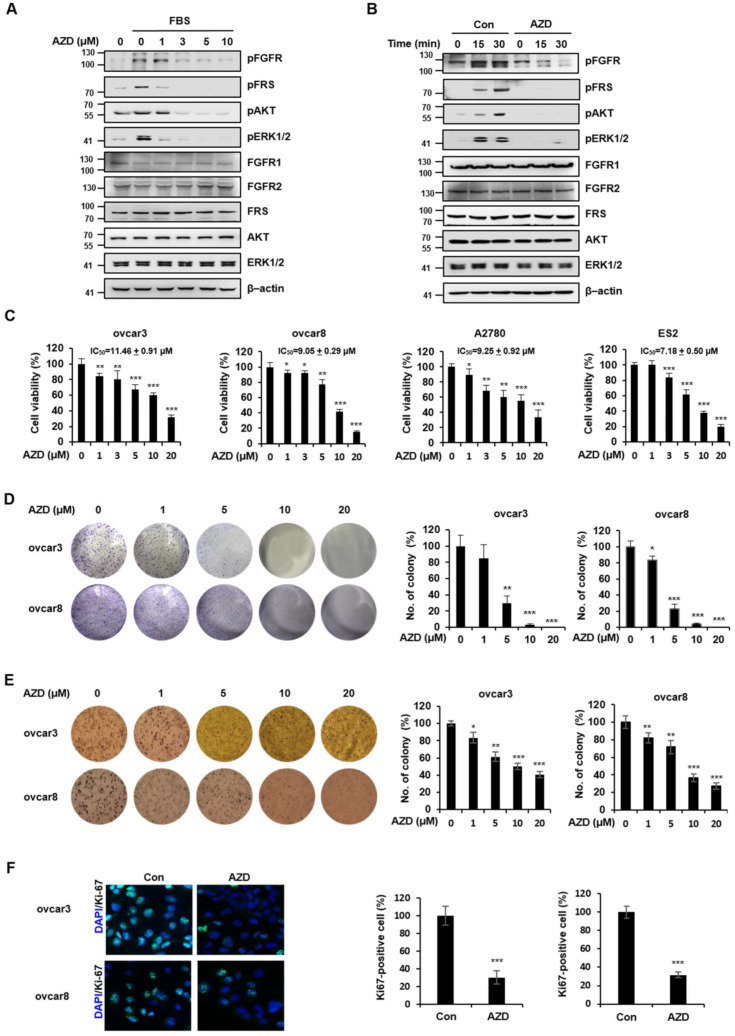
AZD4547 exerts antiproliferative effects in ovarian cancer cells. (**A**) Ovcar3 cells were pretreated with AZD4547 (0, 1, 3, 5, and 10 µM) for 2 h, followed by fetal bovine serum (FBS) treatment. (**B**) Ovcar8 cells were pretreated with 10 µM AZD4547 for 2 h, then stimulated by FBS for the indicated times. (**C**) Ovcar3, ovcar8, ES2, and A2780 cells were treated with AZD4547 for 48 h. Cell viability was determined using the CCK-8 assay. (**D**) Ovcar3 and ovcar8 cells were treated with AZD4547 for 1 week. (**E**) Ovcar3 and ovcar8 cells were seeded onto soft agar and treated with AZD4547 for 3 weeks. Left panel, representative images of colony formation. Right panel, quantification of the corresponding colony formation. (**F**) Ovcar3 and ovcar8 cells were treated with 5 µM AZD4547 for 24 h. Left panel, representative images of DAPI/Ki-67 immunofluorescence. Right panel, quantification of the corresponding immunofluorescence. FGF, fibroblast growth factor; FGFR, fibroblast growth factor receptor; FRS, fibroblast growth factor receptor substrate; ERK1/2, extracellular signal-regulated kinase 1/2; AZD, AZD4547. * *p* < 0.05, ** *p* < 0.01, *** *p* < 0.001.

We examined the inhibitory effect of AZD4547 on FGF/FGFR signaling in ovcar3 ovarian cancer cells. The FGF/FGFR signaling pathway was activated by fetal bovine serum (FBS), as evidenced by the elevated phosphorylation levels of FGFR, FGFR substrate (FRS), and extracellular signal-regulated kinase 1/2 (ERK1/2) (Figure 2A). The AZD4547 treatment dose-dependently attenuated the phosphorylation levels of FGFR, FRS, and ERK1/2, which demonstrated the inhibitory effect of AZD4547 on the FGF/FGFR signaling pathway.

Because FGR/FGFR signaling is a well-established signaling pathway that leads to uncontrollable cell growth [8], we next assessed the cytotoxic and antiproliferative effects of AZD4547 in various ovarian cancer cell lines. As shown in Figure 2B, AZD4547 decreased the viability of ovcar3, ovcar8, ES2, and A2780 cells in a dose-dependent manner. To further confirm the antiproliferative effect of AZD4547, we performed an anchorage-dependent colony formation assay and found that AZD4547 significantly decreased the number of ovcar8 cell colonies (Figure 2C). Cancer cells are capable of growing without attachment to substrates [23]. To assess the inhibitory effect of AZD4547 on the anchorage-independent growth of cancer cells, we performed a soft agar colony formation assay (Figure 2D). We observed that AZD4547 also inhibited anchorage-independent colony formation in ovcar3 and ovcar8 cells.

Lastly, we validated the antiproliferative effect of AZD4547 by determining the immunofluorescence level of Ki-67, a marker that is associated with cell proliferation. As shown in Figure 2E, the immunofluorescence intensity of Ki-67 was significantly decreased in the AZD4547-treated group compared with that in the control group, thereby confirming the antiproliferative effect of AZD4547 in ovarian cancer cells.

### 2.3. AZD4547 Induces Apoptosis in Ovarian Cancer Cells

**Figure 3:**
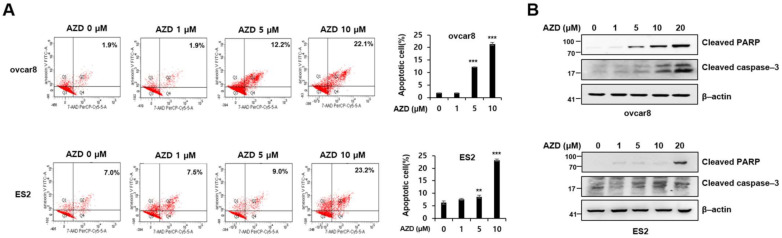
AZD4547 induces apoptosis in ovarian cancer cells. (**A**) Ovcar3 and ES2 cells were pretreated with AZD4547 (0, 1, 5, and 10 µM) for 48 h and stained with annexin V and 7-AAD. Left panel, representative figures of three independent experiments. Right panel, quantification of the corresponding group. (**B**) Western blot analyses of AZD4547-treated ovcar8 and ES2 cells. AZD, AZD4547. ** *p* < 0.01, *** *p* < 0.001.

Because AZD4547 might exert its antiproliferative effect via the induction of apoptosis [24], we investigated whether AZD4547 induces apoptosis in ovarian cancer cells. We found that AZD4547 significantly increased the population of apoptotic cells among ovcar8 and ES2 cells in a dose-dependent manner (Figure 3A). Western blot analysis also revealed increased levels of cleaved PARP and cleaved caspase-3 in both the ovcar8 and the ES2 cells (Figure 3B), which confirmed the apoptosis-inducing effect of AZD4547 as a possible mechanism of the antiproliferative effect of AZD4547.

### 2.4. AZD4547 Inhibits the Migration and Invasion of Ovarian Cancer Cells

**Figure 4:**
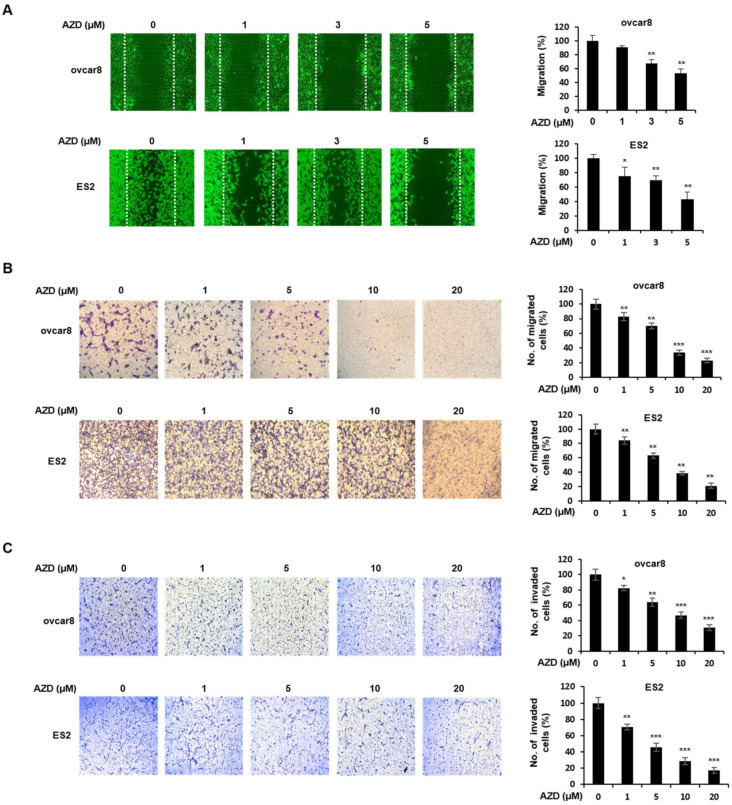
AZD4547 inhibits the migration and invasion of ovarian cancer cells. (**A**) Ovcar8 and ES2 cells were treated with thymidine and AZD4547 for 30 h. White lines indicate the wound borders at the beginning of the assay. Left panel, representative figures from three independent experiments. Right panel, quantification of the percentage of cell-covered area of the corresponding experimental group. (**B**,**C**) Ovcar8 and ES2 cells were treated with AZD4547 for 24 h. Cell migration (**B**) and invasion (**C**) were determined by transwell assay. Left panel, representative images of migrated cells. Right panel, quantification of migrated and invaded cells in the corresponding group. AZD, AZD4547. * *p* < 0.05, ** *p* < 0.01, *** *p* < 0.001.

Ovarian cancer is a highly metastatic disease, and more than 70% of patients have metastases [25]. Metastasis is a multistep process in which the migration and invasion of cancer cells are critical steps [26]. A previous study showed that the FGR/FGFR pathway plays an important role in the migration of cancer cells [8]. Thus, we assessed whether AZD4547 inhibits the migration and invasion of ovarian cancer cells using wound-healing and transwell assays (Figure 4A–C). AZD4547 inhibited the migration of ovarian cancer cells in a dose-dependent manner. The inhibitory effect of AZD4547 on cell migration and invasion was also confirmed in Figure 4B,C, which shows that AZD4547 significantly mitigated the migration and invasion of the ovarian cancer cells.

### 2.5. AZD4547 Impedes the Formation of Cancer Stemness in Ovarian Cancer Cells

**Figure 5:**
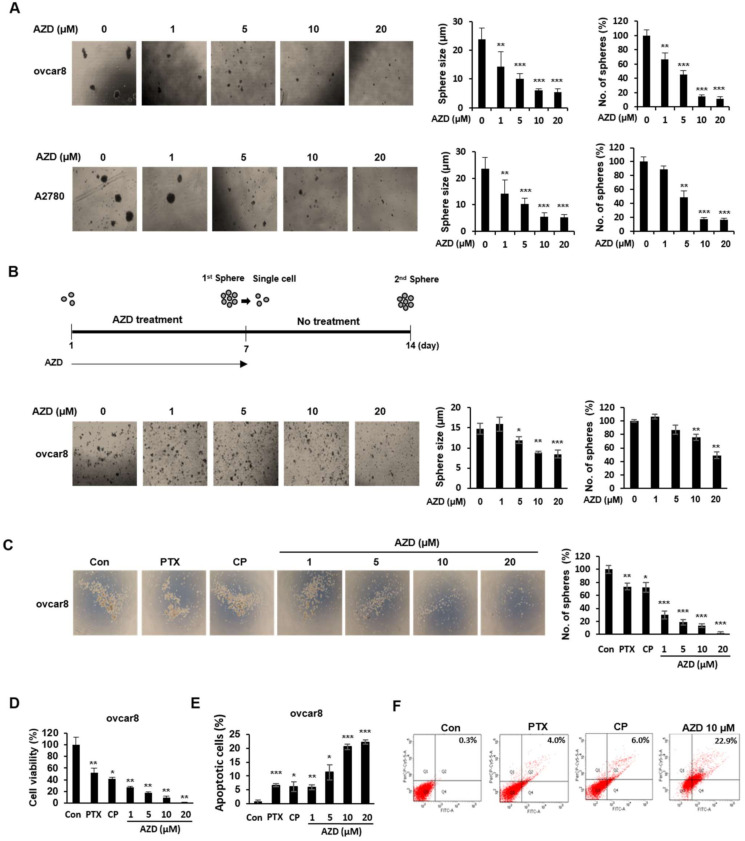
AZD4547 impedes the cancer stemness characteristics of ovarian cancer cells. (**A**) Cells were seeded into ultra-low-attachment 6-well plates and treated with AZD4547 for 7 days. Left panel, representative images of sphere-forming cells. Right panel, quantification of the size and number of sphere-forming cells. (**B**) Spheroid cells were collected and dissociated into single cells. Single cells were again seeded into ultra-low-attachment 6-well plates and cultured without AZD treatment for 7 days. Left panel, representative images of sphere-forming cells. Right panel, quantification of the size and number of sphere-forming cells. (**C**) Ovcar8 cells were seeded into ultra-low-attachment 6-well plates and treated with paclitaxel (PTX, 5 nM), cisplatin (CP, 10 µM), and AZD4547 (0, 1, 5, and 10 µM) for 7 days. Left panel, representative images of sphere-forming cells. Right panel, quantification of the size and number of sphere-forming cells. (**D**) Ovcar8 cells were treated with AZD4547 for 7 days, then cell viability was determined using the CCK-8 assay. (**E**,**F**) Apoptosis of ovcar8 cells were determined by FACS analysis. PTX, paclitaxel; CP, cisplatin; AZD, AZD4547. * *p* < 0.05, ** *p* < 0.01, *** *p* < 0.001.

Cancer stem cells (CSCs) are a small subpopulation of cells within tumors with capabilities of self-renewal, differentiation, and tumorigenicity [27]. We explored whether AZD4547 affects cancer stemness in ovarian cancer cells. To test our hypothesis that AZD4547 might inhibit the stemness characteristics of ovarian cancer cells, we performed a sphere-formation assay in ovcar8 and A2780 cells and found that AZD4547 markedly decreased the size and number of the spheroids (Figure 5A). To further test our hypothesis, we investigated the effect of AZD4547 on the self-renewal abilities of CSCs and observed that AZD4547 decreased the size and number of the secondary spheroids (Figure 5B). Subsequently we determined the extent of the inhibitory effect of AZD4547 on cancer stemness by comparing spheroid formation in AZD-treated ovcar8 cells with that in paclitaxel (PTX)- and cisplatin (CP)-treated ovcar8 cells (Figure 5C). Figure 5C clearly shows that the inhibitory effect of AZD4547 on sphere formation was greater than that of PTX and CP. Accordingly, the cytotoxic and apoptosis-inducing effects of AZD4547 are more profound than those of PTX and CP (Figure 5D–F).

### 2.6. AZD4547 Inhibits Angiogenesis

**Figure 6:**
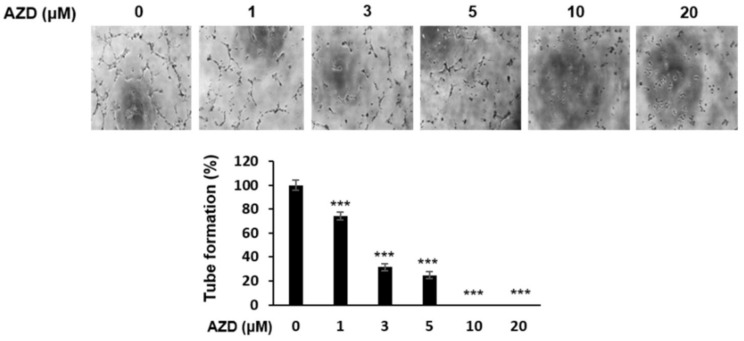
AZD4547 inhibits angiogenesis. Human umbilical endothelial cells were plated into Matrigel-covered wells and treated with AZD4547 for 2 h, followed by serum treatment, to stimulate tube formation. Upper panel, representative images of tube formation. Lower panel, quantification of tube formation. AZD, AZD4547. *** *p* < 0.001.

Because FGFs are also known to be representative angiogenic growth factors [28], we investigated the antiangiogenic effect of AZD4547 in HUVECs. As shown in Figure 6, the treatment of HUVECs with AZD4547 noticeably decreased the number of tube bridges compared with that in the control group. These results suggest that AZD4547 exerts an antiangiogenic effect.

### 2.7. AZD4547 Hinders In Vivo Tumor Formation

**Figure 7:**
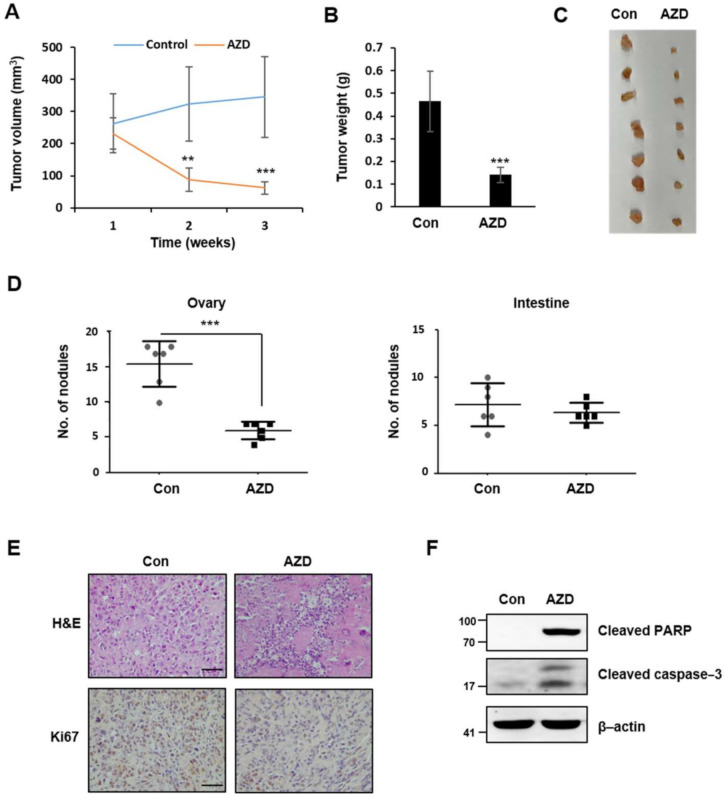
AZD4547 hinders in vivo tumor formation and metastasis. (**A**–**C**) Ovcar8 cells (1.0 × 10^7^ cells) were transplanted subcutaneously (s.c.) into mice. The average tumor volume (**A**) and weight (**B**) of the ovcar8 cell-derived tumors from the control and AZD4547 (15 mg/kg)-treated mice was measured. (**D**) In the ovarian peritoneal metastasis model, SK-OV3-i.p. cells (1.0 × 10^7^ cells) were intraperitoneally (i.p.) injected into nude mice. The number of metastases was determined in the control and AZD4547 (15 mg/kg)-treated mice. (**E**) Images of hematoxylin and eosin and Ki67 staining of tumor tissues in s.c. xenograft mice (scale bar 50 µm). (**F**) Western blot analyses of apoptotic markers in tumor tissues obtained from s.c. xenograft mice. AZD, AZD4547. ** *p* < 0.01, *** *p* < 0.001.

To investigate whether the in vitro antitumor effects of AZD4547 are also present in an in vivo model, we subcutaneously injected mice with ovarian cancer cells (1.0 × 10^7^ cells/mice). We recorded the body weight of the mice from the start to the end of the experimental period and found no difference in body weight, treatment-related death, and abnormal behaviors among the control and experimental groups (data not shown), which implies that the chronic administration of AZD4547 was well-tolerated. Mice injected with the vehicle control developed visible tumors, whereas mice treated with AZD4547 (15 mg/kg) exhibited decreased tumor volume and weight (Figure 7A–C). In addition, to determine the effect of AZD4547 on tumor metastasis, we intraperitoneally injected ovarian cancer cells into mice. Consistent with the in vitro invasion and migration results, AZD4547 treatment in vivo led to a decreased number of nodules in an ovarian peritoneal metastasis mouse model (Figure 7D). Finally, immunohistochemistry and Western blot analyses revealed impaired cell proliferation and increased apoptotic features in tumor tissues obtained from the AZD4547-treated mice (Figure 7E,F).

## 3. Discussion

In this study, we examined the antitumor effects and determined the mechanistic details of AZD4547 in ovarian cancer cells. We first demonstrated that AZD4547 suppressed the proliferation and survival of various ovarian cancer cells. In addition, we observed that AZD4547 suppressed the migration and invasion of ovarian cancer cells. Lastly, we presented the intriguing result that AZD4547 noticeably inhibited the stemness of the ovarian CSCs. AZD4547 decreased the number and size of the spheres to a greater extent than PTX and CP, which are first-line chemotherapeutic agents in the treatment of ovarian cancer. CSCs are widely regarded to be responsible for resistance to chemotherapy and radiotherapy [27]. In our study, the ovarian CSCs exhibited resistance to PTX and CP, whereas AZD4547 effectively reduced the stemness properties and survival rate of ovarian CSCs, suggesting that using AZD4547 to target FGF/FGFR signaling is a promising strategy for the elimination of CSCs in ovarian tumors. In addition, we also confirmed the antitumor activity of AZD4547 in vivo, using mouse xenograft models, suggesting the potential of AZD4547 for the clinical application. AZD4547 efficiently inhibited tumor growth and metastasis at a low concentration in vivo (15 mg/kg), given that IC_50_ of AZD4547 on cell proliferation is relatively high in vitro (IC_50_ = 7.18~11.46 µM). We speculate that anti-metastatic and anti-angiogenic effects of AZD4547 might be responsible for the in vivo efficacy of AZD4547 at a low concentration. Furthermore, several clinical trials of AZD4547 revealed that AZD4547 is well-tolerated and shows a good pharmacokinetics in the human body [29,30,31,32], supporting the potential usage of AZD4547 as a chemotherapeutic drug.

Aberrant regulation of FGF/FGFR signaling is considered an oncogenic signaling pathway. Dysregulation of FGF/FGFR signaling has been reported to occur in various types of cancers [28]. Accordingly, numerous studies have evaluated the FGFR signaling pathway as a promising therapeutic target in various types of cancers [33]. Moreover, a plethora of small-molecule inhibitors targeting FGFRs have been developed [28]. FGF401, an FGFR4 inhibitor, was evaluated in a phase I/II clinical trial for hepatocarcinoma [34]. The FGFR1 and FGFR3 inhibitor BGJ398 was investigated in a phase II clinical trial for FGFR1-amplified lung cancer and FGFR1/2-amplified and FGFR3-mutant breast cancer [35]. Erdafitinib, a pan-FGFR inhibitor, has completed clinical trials for urinary carcinoma, glioblastoma, and endometrial cancer and was recently approved for the treatment of metastatic urothelial carcinoma by the US Food and Drug Administration [36].

To date, there is a lack of biomarkers or diagnostic tools for the early detection of ovarian cancer. Moreover, its therapeutic options, other than surgery and chemotherapy, are limited [37,38]. Recent studies revealed that FGFR1 and FGFR2 are amplified in, and are associated with, the chemotherapy sensitivity of ovarian cancer [17,39]. Another study revealed that the higher expression of FGFR4 is correlated with a poorer overall survival rate in patients with ovarian cancer [18]. In this study, we analyzed the expression levels of FGFR1-4 using the open data of the microarray dataset of 744 ovarian cancer tissues and 46 non-paired normal ovarian tissues. FGFR2 and FGFR3 are highly expressed in ovarian cancer tissues and their expression level is correlated with the poor survival outcome of patients with ovarian cancer. However, we could not observe a significant difference in FGFR4 expression between normal and cancer tissues, whereas the previous study reported the upregulation of FGFR4 in ovarian cancer tissues compared to the adjacent normal tissues [18]. This discrepancy might be due to the difference in the sample number and in the criteria for sample selection between the two studies. Nonetheless, the survival analysis of the ovarian cancer population showed consistent results with the previous study, showing that patients with a high expression of FGFR4 presented a poor survival rate. These results collectively indicate that targeting FGF/FGFR signaling may be promising in relation to the development of more efficacious chemotherapeutic agents against ovarian cancer.

The response rates to the selective inhibitors of FGFR vary substantially depending on the type of FGFR aberration and cancer. Cancers with FGFR1 amplification have shown low response rates to AZD4547. Among four patients with FGFR1-amplified non-small-cell lung carcinoma, only one demonstrated partial responses. Patients with FGFR3-aberrant urothelial cancer also showed an uncertain response to this drug [40]. Among twenty patients with FGFR3-mutated bladder cancer, two demonstrated stable responses to AZD4547. In contrast to FGFR1 amplification and FGFR3 mutation, research has shown that the response to AZD4547 among FGFR2-amplified cancers is strong. Among nine patients with FGFR2-amplified gastric cancer, three demonstrated a long-term response to AZD4547 [40]. Tumors carrying the aberration of FGFR fusions also showed high response rates to the inhibition of FGFR. Patients with FGFR2-BicC family RNA-binding protein 1-combined hepatocellular carcinoma showed decreased tumor volumes [41].

Based on the results of the current study, which demonstrated the potential therapeutic effect of AZD4547 in ovarian cancer, and the above-referenced analyses of the AZD4547 clinical trials, which revealed various responses to AZD4547 depending on the type of FGFR aberration, we propose that further studies in which the antitumor effects of AZD4547 are more precisely dissected according to the status of FGFR mutation are warranted for the development of targeted and efficacious therapies for ovarian cancer.

In summary, this study demonstrated the antitumor effect of AZD4547 on ovarian cancer. AZD4547 downregulated FGF/FGFR signaling in ovarian cancer cells, resulting in the suppression of their proliferation and migration. We further demonstrated that AZD4547 effectively targeted ovarian CSCs and markedly inhibited angiogenesis in HUVECs. These in vitro results were recapitulated in vivo in a mouse xenograft model. Taken together, these findings suggest that targeting FGF/FGFR signaling by AZD4547 has significant therapeutic potential for the treatment of ovarian cancer.

## 4. Materials and Methods

### 4.1. Cell Culture and Reagents

Human ovary cancer cell lines, ovcar3, ovcar8, ES2, and A2780, were obtained from the American Type Cell Culture (ATCC, Rockville, MD, USA). All cells were maintained in RPMI 1640 culture media with 10% FBS, 1% antibiotic-antimycotic at 37 °C in an incubator with 5% CO_2_ atmosphere. AZD4547 was purchased from APExBIO (Houston, TX, USA).

### 4.2. Western Blot Analysis

The harvested cells were lysed in RIPA buffer (Thermo Scientific, Boston, MA, USA) containing protease inhibitors and phenylmethylsulfonyl fluoride (Thermo Scientific, Boston, MA, USA). The lysates were centrifuged for 20 min at 7500 rpm at 4 °C and the supernatant was collected. The lysates were quantified using the BCA Protein Assay Kit (Thermo Scientific, Boston, MA, USA). Equal amounts of protein (30 μg) were separated using sodium dodecyl sulphate polyacrylamide gel electrophoresis (SDS-PAGE) and transferred to nitrocellulose membranes (GE Healthcare, Little Chalfont, UK). The membranes were blocked in 5% skim milk in TBS-T (10 mmol/L Tris-HCl, 50 mmol/L NaCl, and 0.25% Tween-20) for 1 hour at room temperature. The membranes were incubated with anti-phospho-FGFR, anti-phospho-FRS, anti-phospho-ERK1/2, anti-pan ERK1/2 (Cell Signaling Technology, Danvers, MA, USA), anti-FGFR1 (Proteintech, Rosemont, IL, USA), anti-FGFR2, anti-FGFR3, anti-cleaved caspase-3, anti-cleaved PARP (Abcam, Cambridge, UK), anti-FRS and anti-β-actin (Santa Cruz Biotechnology, Dallas, TX, USA) antibodies overnight at 4 °C. The membranes were washed in TBS-T and incubated with horseradish peroxidase-conjugated (HRP) secondary antibodies (Santa Cruz Biotechnology, Dallas, TX, USA). Antibody-bound proteins were detected using LAS-3000 (Fujifilm, Tokyo, Japan) with Pierce ECL Western Blotting Substrate (Thermo Fisher Scientific, Boston, MA, USA).

### 4.3. Cell Cytotoxicity Assay

The cells were seeded in 96-well plates (1 × 10^4^ cells/well). The cells were treated with AZD4547 for 48 h. Cell viability was measured using the Cell Counting Kit 8 (CCK-8, Dogindo, Nagasaki, Japan) according to the manufacturer’s instructions. Briefly, CCK-8 reagent was added to the cells for 1 hour, then absorbance was measured at 450 nm using a microplate reader (BioTek, Männedorf, Switzerland) [42].

### 4.4. Colony Formation and Soft Agar Colony Formation Assay

The ovarian cancer cells were seeded in 6-well plates (1 × 10^4^ cells/well). Cells were treated with AZD4547 for 1 week. The cells were fixed in 4% paraformaldehyde (PFA) and stained with 0.1% crystal violet (in 75% ethanol). The colonies for each sample were counted and images of the stained colonies were visualized with a microscope.

Soft agar colony assay was performed in 6-well plates. The bottom layer consisted of 0.6% agarose and the top agar layer consisted of 0.3% agarose. 1 × 10^4^ cells were seeded to the top layer. The cells were treated with AZD4547 for 3 weeks. The colonies were stained using nitro-tetrazolium blue chloride (Sigma Aldrich, St. Louis, MO, USA) for 24 h. The colonies were visualized using a microscope counted using Image J software (NIH, Bethesda, MD, USA).

### 4.5. Immunofluorescent Staining

The ovarian cancer cells were seeded in an 8-well chamber (1 × 10^4^ cells/well). The cells were fixed with 4% PFA for 30 min at room temperature. Permeabilization was conducted with 0.1% Triton X-100, and non-specific binding was blocked with 1% bovine serum albumin (BSA). Staining was performed using the primary anti-Ki-67 antibody (Abcam, Cambridge, UK). Alexa Fluor 488-conjugated rabbit IgG (Invitrogen, Carlsbad, CA, USA) was used as the secondary antibody. Ki-67 expression was analyzed using the Carl Zeiss LSM5 EXCITER fluorescence microscope (Carl Zeiss, Oberkochen, Germany).

### 4.6. Fluorescence-Activated Cell Sorting (FACS)

The cells were seeded in 60 mm plates (1 × 10^4^ cells/plate). The cells were treated with AZD4547 for 48 h. The harvested cells were incubated using the FITC Annexin V Apoptosis Detection Kit with 7-AAD (BioLegend, San Diego, CA, USA) according to the manufacturer’s instructions. The population of dead cells was analyzed by FACS Canto II flow cytometer (BD, Franklin, NJ, USA).

### 4.7. Wound-Healing Assay

For wound-healing analysis, the cells were seeded in 6-well plates (1 × 10^5^ cells/well). When the cells were confluent, the cells were wounded with sterile pipette tips, followed by treatment with AZD4547 for 30 h. To exclude the effect of AZD4547 on cell proliferation, the cells were co-treated with 500 μM thymidine. The migrated cells were visualized using a microscope and analyzed by Image J software.

### 4.8. Migration and Invasion Assay

Cell migration assays were carried out in transwell with an 8 μm chamber (Corning, Tewksbury, MA, USA). For cell invasion assays, the transwell chamber filters were coated with 1 μg/mL Matrigel (Corning, Tewksbury, MA, USA). The 1 × 10^4^ cells resuspended with serum-free media were added to the upper chamber, and the complete media was added to the lower chambers. After 24 h incubation, filters were fixed in 4% PFA for 10 min and stained with 0.1% crystal violet for 30 min. Migrated cells were visualized and counted using a microscope.

### 4.9. Tumor Sphere Formation and Self-Renewal Capacity Assay

Ovarian cancer cells were resuspended in serum-free B-27/Neurobasal media (Thermo Fisher Scientific, Waltham, MA, USA) containing 20 ng/mL EGF; 10 ng/mL bFGF (R&D system, Minneapolis, MN, USA); 2 mM L-glutamine; 20 mM HEPES (Sigma Aldrich, Saint Louis, MO, USA); 2.5 μg/mL amphotericin B (Thermo Fisher Scientific, Waltham, MA, USA); and 1% penicillin/streptomycin. For sphere formation assay, 1 × 10^4^ cells were seeded in ultra-low attachment 6-well plates (Corning, Tewksbury, MA, USA) and treated with AZD4547 for 7 days.

For the self-renewal capacity assay, sphere-forming cells were harvested. In addition, 1 × 10^3^ cells were seeded in ultra-low attachment 6-well plates and grown without AZD4547 for 7 days. The number and size of the spheres were visualized and calculated using a microscope.

### 4.10. Matirgel Tube Formation Assay

Tube formation was performed in 96-well plates with pre-coated Matrigel. HUVECs were plated at a seeding density of 5 × 10^3^ cells/well with a pre-treatment of AZD4547. After 2 h incubation, serum was added to the cells. After 4 h, tube formation was quantified by counting the number of branches of the tubes by using a microscope.

### 4.11. Bioinformatic Analysis Using Public Data and Web Platform

The differential gene expression data between normal ovary and ovarian cancer tissue were obtained from the TNM plotter database (https://TNMplot.com, accessed on 20 December 2020). The TNM plotter is a web platform that provides data comparing gene expression among normal, tumor, and metastatic tissue in various cancer types [43]. Microarray data of each gene in the TNM plotter was obtained from the NCBI Gene Expression Omnibus (GEO) datasets (https://www.ncbi.nlm.nih.gov/geo, accessed on 20 December 2020). Fold change value and the Mann–Whitney p value were provided with a box plot of gene expression of FGFR1, 2, 3, and 4 in non-paired tumor and normal ovarian tissues. A p value less than 0.05 was considered as statistically significant. The survival data of serous ovarian cancer patients were obtained from the KM plotter database (https://KMplot.com, accessed on 20 December 2020). The KM plotter is an online platform that contains the gene expression and survival data of various types of cancer patients [44]. The survival data of serous ovarian cancer in the KM plotter were analyzed from The Cancer Genome Atlas database (https://www.cancer.gov/tcga, accessed on 20 December 2020) and from the following datasets of GEO database: GSE14764, GSE15622, GSE18520, GSE19829, GSE23554, GSE26193, GSE26712, GSE27651, GSE30161, GSE3149, GSE51373, GSE63885, GSE65986, and GSE9891. The association of the progression-free survival of serous ovarian cancer patients and each gene expression of FGFR1, 2, 3, and 4 were visualized in a Kaplan-Meier survival plot. A p value less than 0.05 was considered as statistically significant.

### 4.12. In Vivo Xenograft

F*e*male seven-week-old BALB/c nude mice and NOD/ShiLtJ-Rag2em1AMC Il2rgem1AMC (NRGA) mice were purchased from Jackson Laboratory (Bar Harbor, ME, USA) and maintained in accordance with the institutional guidelines of Keimyung University, School of Medicine. All in vivo studies were carried out according to approved experimental protocols (KM-2020-08R1). The mice were acclimated for 1 week in individually ventilated cages (IVC) under sterile and standardized environmental conditions (25 ± 2 °C room temperature, 50 ± 10% relative humidity, and 12 h light-dark rhythm). Ovcar8 cells (1.0 × 10^7^ cells) were transplanted subcutaneously into the left flank region of the NRGA mice on experimental day zero. Two weeks after xenotransplantation, the mice were intraperitoneally treated with either AZD4547 (15 mg/kg) or DMSO every other day for 3 weeks. Tumor size was measured at the time of injection and once per week afterwards with a caliper in two dimensions. Individual tumor volumes (V) were calculated by the formula V = (length × [width]2)/2.

For the ovarian peritoneal metastasis model, female BALB/c nude mice were injected with 1 × 10^7^ SK-OV3-i.p. cells intraperitoneally. Five weeks after xenotransplantation, the mice were intraperitoneally treated with either AZD4547 (15 mg/kg) or DMSO every other day for 3 weeks. Eight weeks post induction of peritoneal metastasis, the mice were sacrificed to determine the number of metastases.

### 4.13. Immunohistochemistry (IHC)

The mouse tumor tissues were fixed with 10% formalin and embedded in paraffin and sectioned. The sections were stained with H&E for light microscopic examination. For assessment of the Ki-67 staining, 5 μm sections were permeabilized in PBS, incubated in a 10 mM sodium citrate buffer with pH 6.0 for 10 min at 100 °C with polyclonal anti-Ki-67 antibody (Abcam, Cambridge, UK). Sections were then incubated with secondary antibody (1:200 dilution, Santa Cruz Biotechnology, Dallas, TX, USA), followed by staining with diaminobenzidine chromogen (Cat# SK-4105, Vector Laboratories, Burlingame, CA, USA) and counterstaining with hematoxylin (Cat# S3309, Dako, Glostrup, Denmark). The stained sections were examined under microscopy (Nikon, Tokyo, Japan).

### 4.14. Statistical Analysis

All data were generated with at least three independent experiments in duplicate. The data are represented as means ± S.D. (n ≥ 3). The Student’s *t*-test was performed with *p* < 0.05 being considered as statistically significant.

## Abbreviations

CSC	Cancer stem cells
CP	Cisplatin
ERK1/2	Extracellular signal-regulated kinase ½
FGF	Fibroblast growth factor
FGFR	Fibroblast growth factor receptor
HUVEC	Human umbilical vein endothelial cell
PTX	Paclitaxel
